# Sleep state misperception: is there a CNS structural source?

**DOI:** 10.5935/1984-0063.20200039

**Published:** 2021

**Authors:** Giselle de Martin Truzzi, Igor de Lima Teixeira, Lucila Bizari Fernandes do Prado, Gilmar Fernandes do Prado, Sergio Tufik, Fernando Morgadinho Coelho

**Affiliations:** 1 Universidade Federal de São Paulo, Departamento de Psicobiologia - São Paulo - Brazil.; 2 Universidade Federal de São Paulo, Departamento de Neurologia e Neurocirurgia - São Paulo - Brazil.

**Keywords:** Sleep, Toxoplasmosis, Cerebral, Sleep Initiation and Maintenance Disorders, Acquired Immunodeficiency Syndrome

## Abstract

**Introduction:**

We describe a case of sleep state misperception in a patient with a neurotoxoplasmosis lesion in the left nucleocapsular region.

**Case report:**

A 40-year-old female patient presented relating sleeplessness over the past 2 years, concurrent with progressive headaches, dizziness and motor and sensory deficits in the right upper and lower limbs. She had a history of AIDS, on irregular antiretroviral therapy and neurotoxoplasmosis. A polysomnography confirmed the hypothesis of sleep state misperception, and magnetic resonance imaging revealed a residual lesion in the left nucleocapsular region.

**Conclusion:**

Different models consider that the sleep state misperception could be correlated to structural abnormalities of the central nervous system. A recent study showed that the medial prefrontal cortex had a lower activation in patients with unrefreshing sleep due to chronic fatigue syndrome. This case report highlights the possibility of sleep state misperception having - at least partially - an anatomical substrate in the left nucleocapsular region.

## INTRODUCTION

Sleep state misperception (SSM) or paradoxical insomnia is characterized by an underestimation of the sleep time, compared to the real total sleep time (TST) that is established after polysomnography (PSG) or actigraphy^1,2^. Although SSM is a frequent sleep disorder seen in insomniac individuals, it still is difficult to correctly recognize and a challenge to treat^3^.

The SSM pathophysiology is not completely known. SSM is related to changes in the sleep time perception, personality traits, memory and psychological disorders^4,5^. Traumatic brain injuries can also lead to various sleep disorders, including insomnia. Diverse brain lesions can lead to anxiety, depression and may cause pain that contributes to insomnia^6^.

We describe a patient with acquired immune deficiency syndrome (AIDS) with acute SSM after brain damage secondary to neurotoxoplasmosis.

## CASE REPORT

We report the case of a 40-year-old female patient, married, currently unemployed but with an occupational history of working at a gas station, who presented to the clinic reporting sleeplessness over the past two years.

She reported not sleeping at all during most nights. On the nights she perceived to sleep, she would fall asleep around 4:00 a.m. only, and just for a few minutes. Her usual bedtime was 10:00 p.m. and she got up around 9:00 a.m.

The patient informed irritability and recurring negative thoughts worried her all night. She reported excessive daytime sleepiness, but the Epworth Sleepiness Scale score was zero. The patient denied napping during the day, nightmares, snoring, restless leg syndrome symptoms, or other sleep complaints before these last two years.

**Figure 1:**
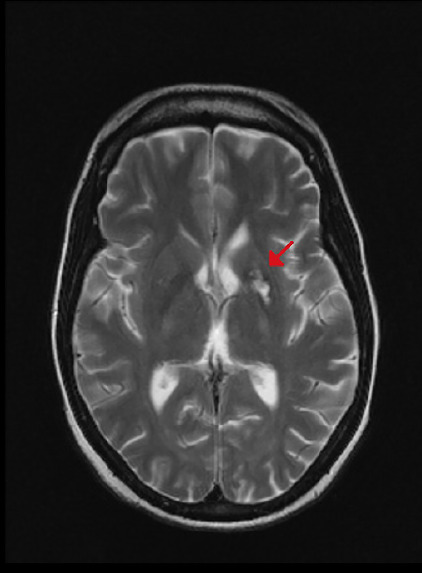
MRI of the brain shows a residual lesion in the left nucleocapsular region (red arrow).

The patient had a history of acquired immune deficiency syndrome (AIDS, or stage IV HIV infection, according to the World Health Organization) that had been diagnosed 13 years previously, during a prenatal care visit. Treatment with an antiretroviral therapy was initiated, but the adhesion to treatment was extremely poor; over the past 2 years, total CD4 count ranged from 15 to 85/µl and HIV-RNA count from 10,560 to 24,343 copies/ml. She also had a previous history of appendectomy with ileostomy, with subsequent diagnosis of diffuse malignant B-cell lymphoma, from which she received appropriate care and is on follow-up. Two years ago, she began episodes of a progressive headache and dizziness. A computed tomography (CT) scan of the brain during the investigation revealed an extensive area of hypodensity in the left nucleocapsular region, showing lesion in ring enhancement in the region with surrounding perilesional edema after contrast administration. A magnetic resonance imaging (MRI) of the brain acquired afterward showed a residual lesion in the left nucleocapsular region (Figure 1). Sulfadiazine, pyrimethamine, folinic acid and dexamethasone were used to treat neurotoxoplasmosis, along with the antiretroviral therapy, with good outcomes.

The patient was referred to the sleep disorders clinic with the complaint of total insomnia. The initial treatment included amitriptyline and trazodone, with no response. The neurological examination was then normal, and no cognitive disorder was identified. A polysomnography showed a sleep efficiency of 74,2%, and total sleep time of 290 minutes (16.4% N1, 36.9% N2, 27.6% N3 and 19.1% REM), 16.6 arousals per hour, waketime after sleep onset of 97.5 minutes, normal apnea-hypopnea index (1.0/h), a nadir SaO2 97% and no indication of periodic limb movements in sleep index of 1.4/h.

The patient, however, reported not being able to sleep at any time during the exam, leading to the diagnosis of SSM. She received the orientation about the disorder and on sleep hygiene and cognitive behavior therapy was recommended.

## DISCUSSION

The prevalence of insomnia in the HIV-seropositive population is higher than in the general population^7^. Although this finding is not fully understood, there are a few studies about HIV and focal brain findings causing insomnia^8^. Authors show that sleep disturbances in HIV patients is higher than in the general population with prevalence about 60%^7^. The overlap between psychiatric symptoms and HIV can be a factor to explain higher prevalence of sleep disorders. However, the HIV patient can show low signs of psychiatric illness with no improvement with antidepressant treatment.

Although there is no correlation between CD4 levels and insomnia, studies demonstrated that patients with most severe disease and prolonged period of diagnosis have higher prevalence of insomnia^7^. The use of drugs to treat HIV, such as efavirenz, can also cause sleep disorders. Interestingly, focal lesions of CNS, in the region involved in sleep regulation, was related to abrupt change in sleep pattern.

Different models consider that the SSM could be correlated to structural abnormalities of the CNS^4^. Brain lesions, such as traumatic brain injury, may drive sleep disorders, such as insomnia and overestimation of insomnia^9,10^. Authors suggested there could be an anatomical relation between insomnia and brain stress ways. Usually, there is a negative balance between ventrolateral preoptic nucleus (VLPO) and monoamine circuits during sleep. In patients with SSM, however, both VLPO and monoamine circuits are simultaneously activated, with a consequent instability of phases during sleep^11,12^. Animal models showed that lesions in the VLPO area led to insomnia and significant decrease in slow-wave sleep^13,14^. The neuronal activation of VLPO is associated with the neuronal activation of the limbic system explaining the tendency of SSM^11,12^.

A recent study showed that the medial prefrontal cortex had a lower activation in patients with unrefreshing sleep due to chronic fatigue syndrome, and there are descriptions of total insomnia and delirium in patients with lacunar stroke of the internal capsule and of the left thalamus^15,16^. The temporal relation between the beginning of the symptoms and the structural damage highlights the possibility of SSM having – at least partially – an anatomical substrate in the left nucleocapsular region.

This case report shows a temporal relationship between brain injury and the onset of SSM. The patient previously had no sleep disorders or sleep complains. SSM is a challenge to diagnosis, treatment and scientific understanding. We agree that many factors can be involved in SSM in this case report, however the temporal association between CNS lesion and initial sleep complain must be related.

## References

[1] van der Walt JP, Johannsen E (1974). The dangeardien and its significance in the taxonomy of the ascomycetous yeasts. J Urol.

[2] Ito E, Inoue Y (2015). International Classification of Sleep Disorders - third edition (ICSD-3). American Academy of Sleep Medicine. Nihon Rinsho.

[3] Choi SJ, Suh S, Ong J, Joo E Y (2016). Sleep misperception in chronic insomnia patients with obstructive sleep apnea syndrome: implications for clinical assessment. J Clin Sleep Med.

[4] Harvey AG, Tang NK (2012). (Mis)perception of sleep in insomnia: a puzzle and a resolution. Psychol Bull.

[5] Bianchi MT, Williams KL, McKinney S, Ellenbogen JM (2013). The subjective-objective mismatch in sleep perception among those with insomnia and sleep apnea. J Sleep Res.

[6] Castriotta RJ, Murthy JN (2011). Sleep disorders in patients with traumatic brain injury: a review. CNS Drugs.

[7] Low Y, Goforth H, Preud’homme X, Edinger J, Krystal A (2014). Insomnia in HIV-infected patients: pathophysiologic implications. AIDS Rev.

[8] Gunnarsdottir KM, Kang YM, Kerr MS, Sarma SV, Ewen J, Allen R (2015). A look at the strength of micro and macro EEG analysis for distinguishing insomnia within an HIV cohort. Conf Proc IEEE Eng Med Biol Soc.

[9] Ouellet MC, Morin CM (2006). Subjective and objective measures of insomnia in the context of traumatic brain injury: a preliminary study. Sleep Med.

[10] Ouellet MC, Savard J, Morin CM (2004). Insomnia following traumatic brain injury: a review. Neurorehabil Neural Repair.

[11] Saper C, Chou TC, Scammell TE (2001). The sleep switch: hypothalamic control of sleep and wakefulness. Trends Neurosci.

[12] Cano G, Mochizuki T, Saper CB (2008). Neural circuitry of stress-induced insomnia in rats. J Neurosci.

[13] Sallanon M, Denoyer M, Kitahama K, Aubert C, Gay N, Jouvet M (1989). Long-lasting insomnia induced by preoptic neuron lesions and its transient reversal by muscimol injection into the posterior hypothalamus in the cat. Neuroscience.

[14] Lu J, Greco MA, Shiromani P, Saper CB (2000). Effect of lesions of the ventrolateral preoptic nucleus on NREM and REM sleep. J Neurosci.

[15] Shan ZY, Kwiatek R, Burnet R, Del Fante P, Staines DR, Marshall-Gradisnik SM (2017). Medial prefrontal cortex deficits correlate with unrefreshing sleep in patients with chronic fatigue syndrome. NMR Biomed.

[16] Pedro P, Telles-Correia D, Godinho I, Chagas C (2015). Onset of psychosis at age 81? With regard to frontal lobe syndromes. Einstein (Sao Paulo).

